# Dynamic changes of gut microbiota and hepatic functions are different among biliary atresia patients after Kasai portoenterostomy

**DOI:** 10.1002/ctm2.728

**Published:** 2022-02-20

**Authors:** Yingchao Li, Cheng Guo, Qian Zhou, Wenkui Dai, Ying Zhang, Muxia Li, Ye Wang, Peipei Wang, Lin Liu, Shuaicheng Li, Lin Zhang

**Affiliations:** ^1^ Department of Pediatric Surgery The Second Hospital of Hebei Medical University Shijiazhuang China; ^2^ Department of Pediatrics The Third Hospital of Hebei Medical University Shijiazhuang China; ^3^ Department of Computer Science City University of Hong Kong Hong Kong China; ^4^ Department of Obstetrics and Gynecology Peking University Shenzhen Hospital Shenzhen China; ^5^ Department of Nutrition and Food Hygiene School of Public Health, Peking University Beijing China

Dear Editor,

Biliary atresia (BA) is a fatal neonatal disease resulting in cholestasis and progressive hepatic failure.^1^ Despite the fact that Kasai portoenterostomy (KP) can restore bile drainage, impaired hepatic functions and even advanced cirrhosis can be detected in most of the patients.^2^ Given the interaction between gut microbiota (GM) and hepatic functions,^3^ this study aimed to assess if BA patients with different pre‐surgery GM had different GM dynamics and hepatic functions following KP.

According to the inclusion criteria (see the Supporting Information), we enrolled 26 BA patients aged 19–105 days whose parents approved KP therapy. Then we collected feces for sequencing of 16S rDNA V3‐V4 regions^4^ at five timepoints (Figure S1A). Before KP, 18 patients had *Bifidobacterium*‐dominated (BD) and eight patients had non‐*Bifidobacterium*‐dominated (NBD) GM structure (Figure 1A,B). In the NBD group, *Bacteroides*, *Rothia*, *Defluviitoga* and *Collinsella* represented higher relative abundance when compared to the BD group (Figure 1A). Additionally, microbial samples in the BD group had significantly lower diversity than the NBD group (Figure 1C). Further analysis indicated that inter‐individual dissimilarity in the BD group was dramatically lower than that in the NBD group (Figure 1D). In addition, the inter‐individual distance between BD and NBD samples was as high as that in the NBD group (Figure 1D). At the phylum level, *Actinobacteria* represented obviously higher relative abundance in microbial samples of the BD group (averaged 92.99% vs. 49.92% in the NBD group) (Figure 1E). Nonetheless, the average level of Bacteroidetes and Firmicutes in the NBD group was 3.54 and 10.98 times as that in the BD group, respectively (Figure 1E). Functional prediction indicated accumulation of amino acid metabolism, depletion of cell motility, cellular processes and signalling in microbial samples of the BD group (Figure S1B). Intravenous antibiotic exposure caused slight but insignificant changes to the GM composition of BD and NBD patients (Figure S1C).

**FIGURE 1:**
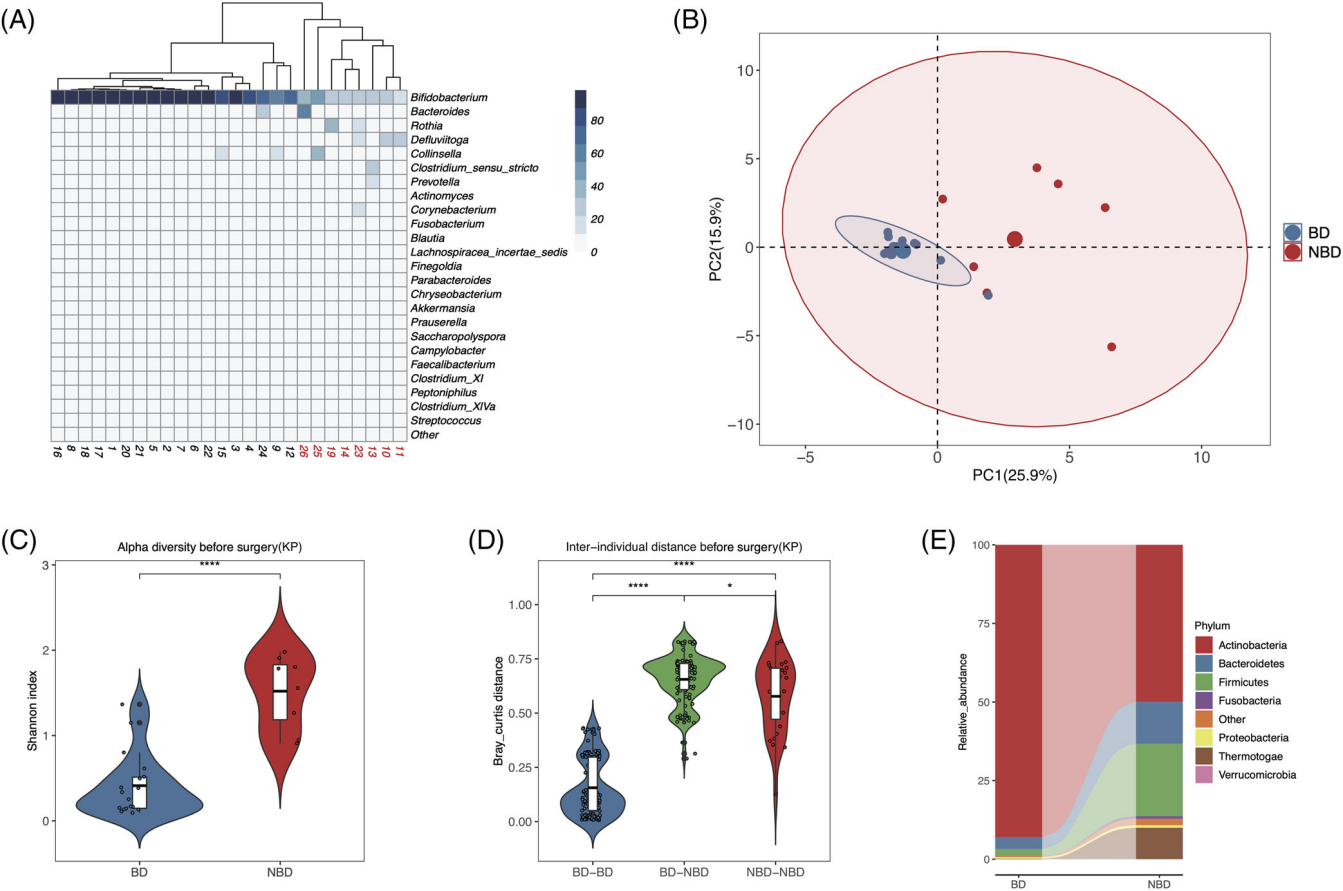
Inter‐individual gut microbiota (GM) differences before surgery. (A) Heatmap of genus‐level microbial components for 26 faecal samples before Kasai portoenterostomy (KP), and sample names in the non‐*Bifidobacterium*‐dominated (NBD) group are labelled red. (B) Principal components analysis of 26 faecal samples before surgery. (C) Alpha‐diversity for *Bifidobacterium*‐dominated (BD) and NBD groups, the Shannon value was (mean ± SD, 0.41 ± 0.37) and (1.52 ± 0.43, *p*‐value < .0001) for BD and NBD groups, respectively. (D) Inter‐sample bray‐curtis distance (dissimilarity) in BD and NBD groups, as well as between BD and NBD groups. (E) Phylum abundance in BD and NBD groups. BD: *Bifidobacterium*‐dominated GM structure before KP; NBD: non‐*Bifidobacterium*‐dominated GM structure before KP. Error bars represent the standard error of the mean. Wilcoxon rank‐sum test was applied to analyse statistical significance for inter‐group differences. *p‐value < .05; ****p‐value  < .0001, respectively

Permutational multivariate analysis of variance (PERMANOVA) unravelled that KP contributed remarkably to post‐surgery GM changes (Figure 2A). Consistently, GM changed significantly after KP (Figure S1D). Inter‐group distance of microbial samples kept high even after KP, which suggested distinct GM dynamics between BD and NBD groups (Figure 2B,C,D). Consistent with transition of *Bifidobacterium*‐dominated GM structure to other GM types following development,^5^ microbial samples in the BD group had higher dynamic changes than those in the NBD group after KP (Figure 2B). A previous study also found a decreased level of *Bifidobacterium* in patients who had clearance of jaundice.^6^ Inter‐individual differences of post‐surgery GM changes in the BD group may be explained by different sensitivities of *Bifidobacterium* strains to bile.^7^

**FIGURE 2:**
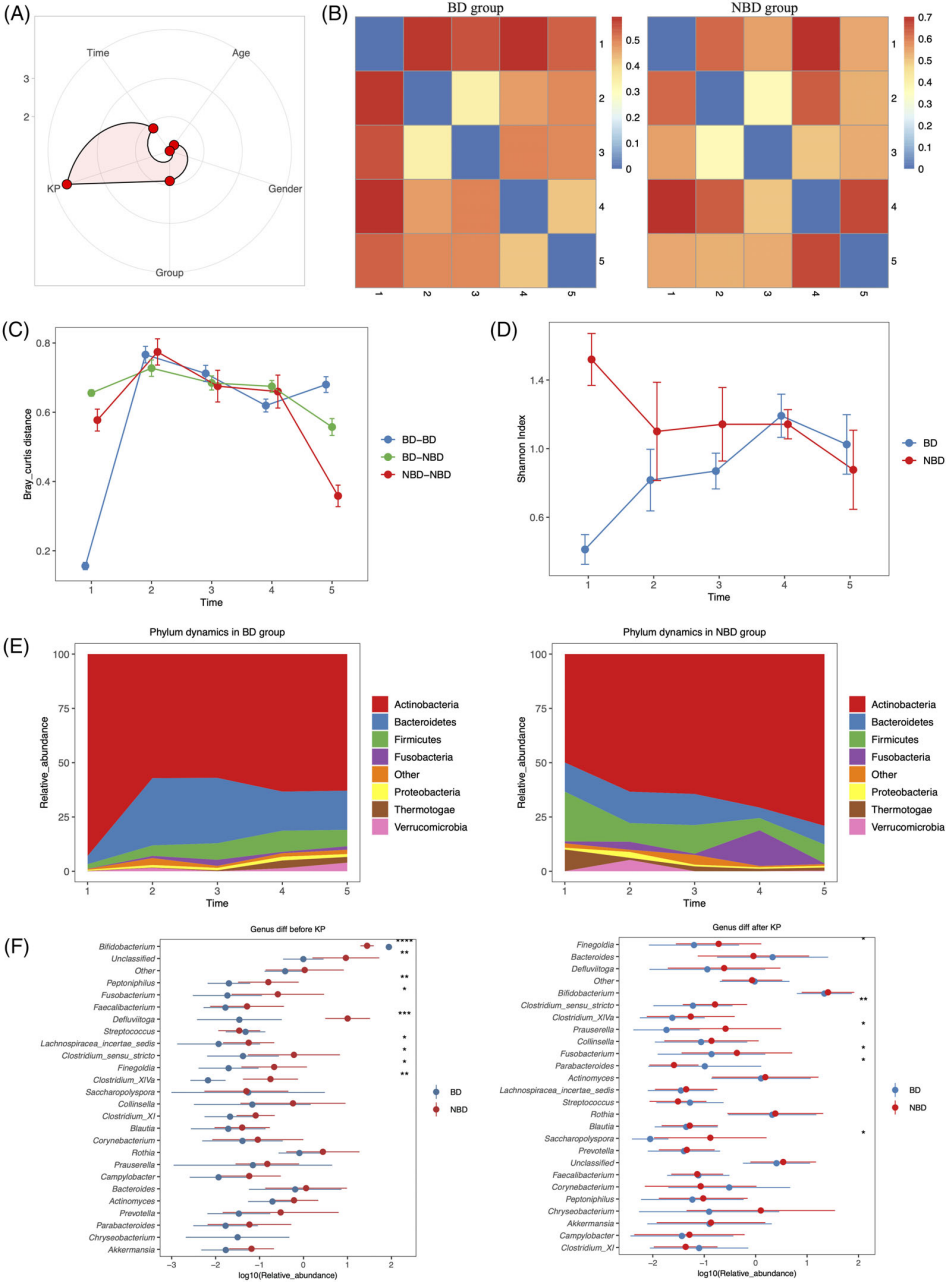
Post‐surgery gut microbiota (GM) dynamics after surgery. (A) PERMANOVA to assess the effects of several indices on GM dynamics: more significant if the point is located near the outer circle. Kasai portoenterostomy (KP) (*p*‐value = .002), time (*p*‐value = 0.044) and pre‐surgery GM structure (“Group” in the figure, *p*‐value = 0.048) contributed remarkably to post‐surgery GM changes. Permutation test was applied to assess the statistical significance. (B) Bray‐curtis distance (dissimilarity) of microbial samples between different timepoints for *Bifidobacterium*‐dominated (BD) and non‐*Bifidobacterium*‐dominated (NBD) groups. (C) Comparison of dynamic bray‐curtis distance (dissimilarity) between microbial samples in BD and NBD groups, respectively, as well as between BD and NBD groups. (D) Alpha diversity of microbial samples at different timepoints for BD and NBD groups. (E) Phylum‐level microbial components at different timepoints for BD and NBD groups. (F) Inter‐group genus differences: the left sub‐figure represented differences before surgery (microbial samples collected before KP), the right sub‐figure represented differences after surgery (all microbial samples collected 1, 3, 7, 30 days after KP), and the importance decreased from top to bottom (e.g., *Bifidobacterium* represents the most importance in inter‐group differences before KP). BD: *Bifidobacterium*‐dominated GM structure before KP; NBD: non‐*Bifidobacterium*‐dominated GM structure before KP. Time 1, 2, 3, 4 and 5 represent the day of hospitalization (before surgery and intravenous antibiotics exposure) 1, 3, 7 and 30 days after surgery, respectively. Error bars represent the standard error of the mean. Wilcoxon rank‐sum test was applied to analyse statistical significance for inter‐group differences. *p‐value < .05, **p‐value < .01, ***p‐value < .001, ****p‐value < .0001

Of the microbial samples in the BD group, phylum *Actinobacteria* levels reduced, while *Bacteroidetes* and *Firmicutes* levels elevated after KP (Figure 2E). There was an opposite trend for dynamic changes of *Actinobacteria*, *Bacteroidetes* and *Firmicutes* in the NBD group (Figure 2E). After surgery, contributions of the bacterial genus in inter‐group differences also changed (Figure 2F). For instance, *Bifidobacterium* was the most robust in contributing to differences between BD (mean ± SD, 88.32 ± 12.87%) and NBD (29.46 ± 10.61%) groups before KP, and was insignificant in explaining inter‐group differences (BD: 37.65 ± 32.31%, NBD: 40.18 ± 31.35%) after therapy (Figure 2F). By contrast, the significance of *Bacteroides* in contributing to inter‐group differences increased after KP (BD: 17.07 ±29.43%, NBD: 9.24 ± 24.00%) (Figure 2F). This possibly suggested normal development of *Bifidobacterium*‐dominated GM to *Bacteroides*‐dominated enterotype.^4^ Additionally, *Defluviitoga* and *Fusobacterium* kept significant contributions to inter‐group differences both before and after KP (Figure 2F).

In the early stage of life, *Bifidobacterium* dominated GM, and depletion of *Bifidobacterium* correlated with a variety of diseases.^5,8^ This may explain better hepatic functions for patients in the BD group before surgery (Figure 3A). PERMANOVA indicated different impacting factors for clinical indicators (Figure 3B). Like GM (Figure 2B), dissimilarity of clinical indicators at different time points was slightly higher in the BD group than in the NBD group (Figure 3C). Despite similar changes following KP, ALT, AST, IBIL levels kept higher in the NBD group and TAB levels kept higher in the BD group (Figure 3D), suggesting the long‐term impact of pre‐surgery GM on hepatic functions. Correlation analysis indicated the association of amino acid metabolism, nucleotide metabolism, membrane transport and signalling molecules and interaction with *Bifidobacterium*, which decreased after KP (Figures 2F and 3E). Nevertheless, environmental adaptation, signal transduction, lipid and energy metabolism correlated with bacterial genus, which had high importance in inter‐group differences after KP, including *Defluviitoga*, *Fusobacterium* and *Bacteroides* (Figures 2F and 3E).

**FIGURE 3:**
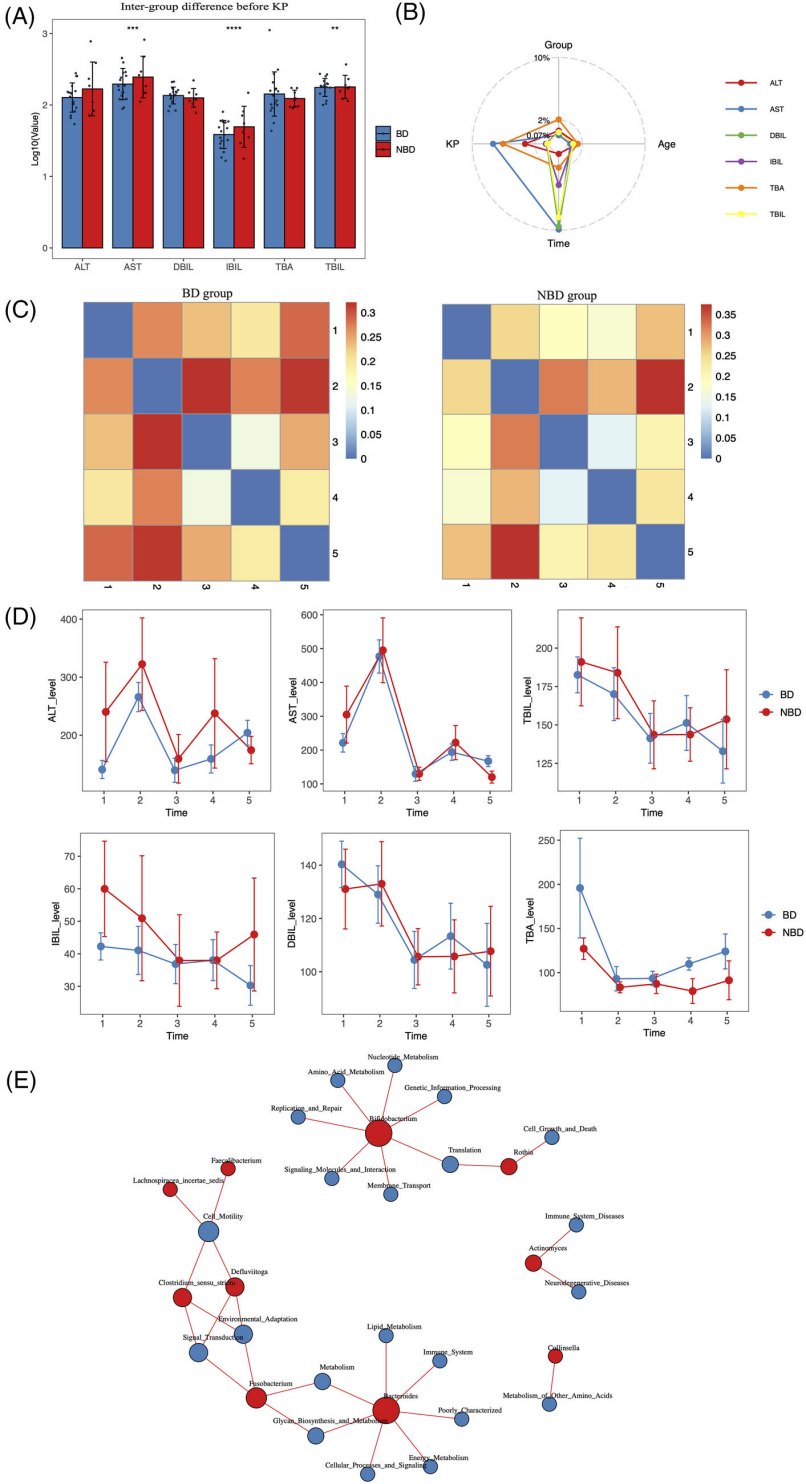
Different dynamic changes of several clinical indicators between *Bifidobacterium*‐dominated (BD) and non‐*Bifidobacterium*‐dominated (NBD) groups. (A) Difference of clinical indicators between BD and NBD groups before surgery. AST, IBIL, and TBIL were statistically increased in NBD patients compared to BD (304.89 ± 237.91 vs. 221.36 ± 115.46 U/L; 59.97 ± 41.48 vs. 42.27±17.69 μmol/L; 190.99 ± 66 vs. 182.56 ± 60 μmol/L in the BD group, respectively), and ALT levels were increased but not statistically significant (240.10 ± 241.99 vs. 140.85 ± 66.09 U/L in the BD group). (B) PERMANOVA to assess the effects of several indices on dynamics of clinical indicators after surgery: more significant if the point is located near the outer circle. Serum levels of AST were impacted by Kasai portoenterostomy (KP) and time most significantly. The TBA level was mainly impacted by KP, and the levels of TBIL, DBIL and IBIL were mainly impacted by time after receiving KP. Permutation test was applied to assess the statistical significance. (C) Bray‐curtis distance (dissimilarity) of clinical indicators between different timepoints for BD and NBD groups. (D) Changes of clinical indicators at different timepoints for BD and NBD groups. (E) Correlation network between bacterial genus and predicted microbial functions. The circle size is positively related with linked edges and the red line represents *r*‐value > .4 and *p*‐value < .05. BD: *Bifidobacterium*‐dominated gut microbiota (GM) structure before KP; NBD: non‐*Bifidobacterium*‐dominated GM structure before KP. Time 1, 2, 3, 4 and 5 represent the day of hospitalization (before surgery and intravenous antibiotics exposure) 1, 3, 7 and 30 days after surgery, respectively. Error bars represent the standard error of the mean. Wilcoxon rank‐sum test was applied to analyze statistical significance for inter‐group differences. **p‐value < .01, ***p‐value < .001, ****p‐value < .0001

Further analysis found negative correlation of *Akkermansia*, *Parabacteroides*, *Actinomyces*, *Fusobacterium* and *Finegoldia* with serum levels of bilirubin, while *Prevotella* correlated negatively with AST and ALT levels in the BD group (Figure S1E). This was partly consistent with prior reports suggesting that *Prevotella* was associated with short fatty acids metabolism and *Akkermansia* was a promising probiotics candidate.^9^ In the NBD group, serum levels of bilirubin, AST and ALT correlated negatively with *Bifidobacterium* and *Collinsella*, but positively correlated with opportunistic pathogens *Saccharopolyspora*, *Corynebacterium* and *Defluviitoga* (Figure S1E). Additional analysis indicated the involvement of clinical indicator‐related microbial genus in the co‐occurrence network (Figure S1F). For example, *Akkermansia* and *Fusobacterium* were in positive correlation in the BD group, while *Saccharopolyspora* and *Defluviitoga* were in positive correlation in the NBD group (Figure S1F). The above‐mentioned correlations implicated the necessity to consider pre‐surgery GM structure when applying prebiotics or probiotics to improve hepatic functions after KP.^10^

Though this study found different dynamic changes of GM and clinical indicators for BA patients who received KP therapy, several limitations should be noted. First, 16S rRNA gene amplicon sequencing only analysed known bacterial genus. Additionally, GM‐derived metabolites were not detected to analyse the interaction between GM and hepatic functions. Second, we only performed sample collection in 30 days after surgery, thus it was impossible to assess long‐term effects of pre‐surgery GM on post‐surgery hepatic functions. Finally, we did not record factors that affected early‐life GM, including delivery mode and feeding pattern. However, lack of information like delivery mode will not affect our findings negatively because the aim was to assess the association of developed GM with hepatic functions.

In conclusion, our study identified different hepatic functions for BA patients with different pre‐surgery GM structures, and emphasised the importance of pre‐surgery GM structures in GM dynamics and restoration of hepatic functions after receiving KP therapy.

## CONFLICT OF INTEREST

The authors declare that they have no conflict of interest.

## Supporting information



Supporting information
