# Supplementary material for: Dynamic changes of gut microbiota and hepatic functions are different among biliary atresia patients after Kasai portoenterostomy
Source: Clin Transl Med. 2022 Feb 20;12(2):e728. doi: 10.1002/ctm2.728 (PMC8858627; doi:10.1002/ctm2.728)
Supplement: Supplementary file 1 — Supporting information [file CTM2-12-e728-s001.pdf]

1.METHODS

1.1. Study design and sample information

Parents of all recruited patients provided informed consents before sample collection. Patients were included following five criteria: (1). younger than one year old; (2). jaundice with dark-colored urine or light-colored stool; (3). the serum level of direct bilirubin (DBIL) > 17.1 μmol/L; (4). hepatic sonographic showed the triangular cord sign, abnormal gall bladder morphology, lack of gall bladder contraction after oral feeding, non-visualization of the common bile duct; (5). hepatobiliary radionuclide imaging: there was non-visualization of the gallbladder and small intestinal activity within 24 hours. According to the inclusion criteria, 36 children with cholestasis were collected from December, 2019 to September, 2021. Thirty-two cases of BA were diagnosed by the intraoperative cholangiography and hilar bile duct exploration, including 26 cases whose parents approved KP therapy. Selected patients all received intravenous antibiotics 1-2 days before and the first 7 days after KP to prevent infections. Time 1, 2, 3, 4, and 5 represent the faecal samples collected on the day of hospitalization (before surgery and intravenous antibiotics exposure), 1, 3, 7 and 30 days after surgery respectively. Besides to five sampling timepoints, we also collected feces sample the day of surgery (1-2 days after intravenous antibiotics exposure) and compared the GM variations after intravenous antibiotics exposure. We also recorded several clinical indicators which represented bile acid metabolism and hepatic functions, including serum levels of total bilirubin (TBIL), DBIL, indirect bilirubin (IBIL), total bile acid (TBA), aspartate transaminase (AST) and alanine transaminase (ALT). A total of 10 patients had cholangitis (3 belonged to NBD group and 7 belonged to NBD group). Twenty patients survived until we prepared this article, and six patients died for different reasons (two belonged to NBD group and four belonged to BD group; two died for severe pneumonia 2 and 4 months after surgery respectively, three die for unclear reason 15, 17 and 23 months after surgery, one died for hepatic failure 7 months after KP).

1.2. DNA extraction, library construction and sequencing

Microbial DNA was extracted by Dneasy PowerSoil Pro Kit (Qiagen, German). Then we amplified16S rDNA V3-V4 hypervariable regions of 16S rRNA gene via primers 338F and 806R, using the PCR kit (TransGenAP221-02, Peking). The quality of PCR products was determined (Qubit, Thermo Fisher Scientific, Singapore) to prepare for library construction (TruSeq DNA PCR-Free kit, Illumina, San Diego, CA, United States). The qualified libraries were sequenced based on Illumina Miseq platform (Illumina, San Diego, CA, United States).

1.3. Data analysis and visualization

Sequencing reads were filtered and annotated as previously reported (reference 4 in the main text). Data was tested for normality using Shapiro-Wilk test and QQ-plot before further analysis. Functional prediction of microbial components was conducted using PICRUSt with default parameters. Random forest classifier was utilized to assess the importance of microbial genus in inter-group differences (package "mlr" in R). Wilcoxon rank-sum test was applied to analyze differences of GM structures, diversity and clinical indicators. The impact of different indices (e.g., gender, age, KP, time and pre-surgery GM structure) on GM distributions was assessed by Permutational Multivariate Analysis of Variance (PERMANOVA) with 9,999 permutations (package "vegan" in R). Correlation between GM and clinical indicators was conduct by package

“Hmisc” in R, and microbial co-occurrence network was analyzed via “psych” and “igraph” packages in R software. Analysis results were visualized using R software.

FIGURE S1. (A) Flowchart of this study. (B) Differences of predicted microbial functions between BD and NBD group before surgery. (C) GM components before and after intravenous antibiotics exposure. (D) Dynamic changes of bacterial genus at different timepoints. Time 1, 2, 3, 4, and 5 represents the day of hospitalization (before intravenous antibiotics exposure), 1, 3, 7 and 30 days after surgery respectively. (E) Correlation between GM and clinical indicators for BD and NBD group. We applied t-test to analyze statistical significance. (F) Microbial co-occurrence network for BD and NBD group. BD: Bifidobacterium-dominated GM structure before KP; NBD: non-Bifidobacterium-dominated GM structure before KP. p-value: *, **, *** and **** represent <0.05, <0.01, <0.001 and <0.0001 respectively.

A

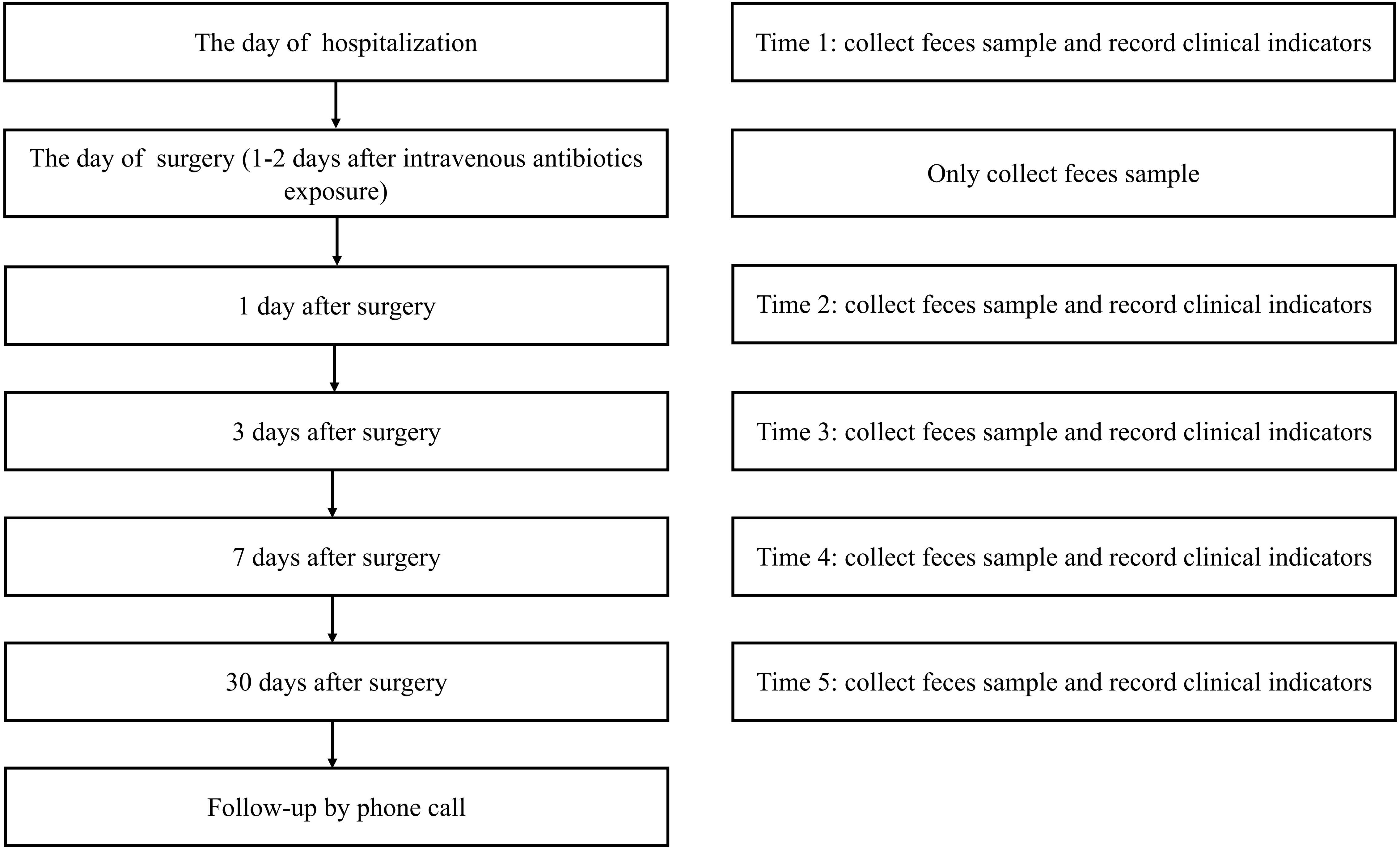
The day of hospitalization
Time 1: collect feces sample and record clinical indicators
The day of surgery (1-2 days after intravenous antibiotics exposure)
Only collect feces sample
1 day after surgery
Time 2: collect feces sample and record clinical indicators
3 days after surgery
Time 3: collect feces sample and record clinical indicators
7 days after surgery
Time 4: collect feces sample and record clinical indicators
30 days after surgery
Time 5: collect feces sample and record clinical indicators
Follow-up by phone call

B

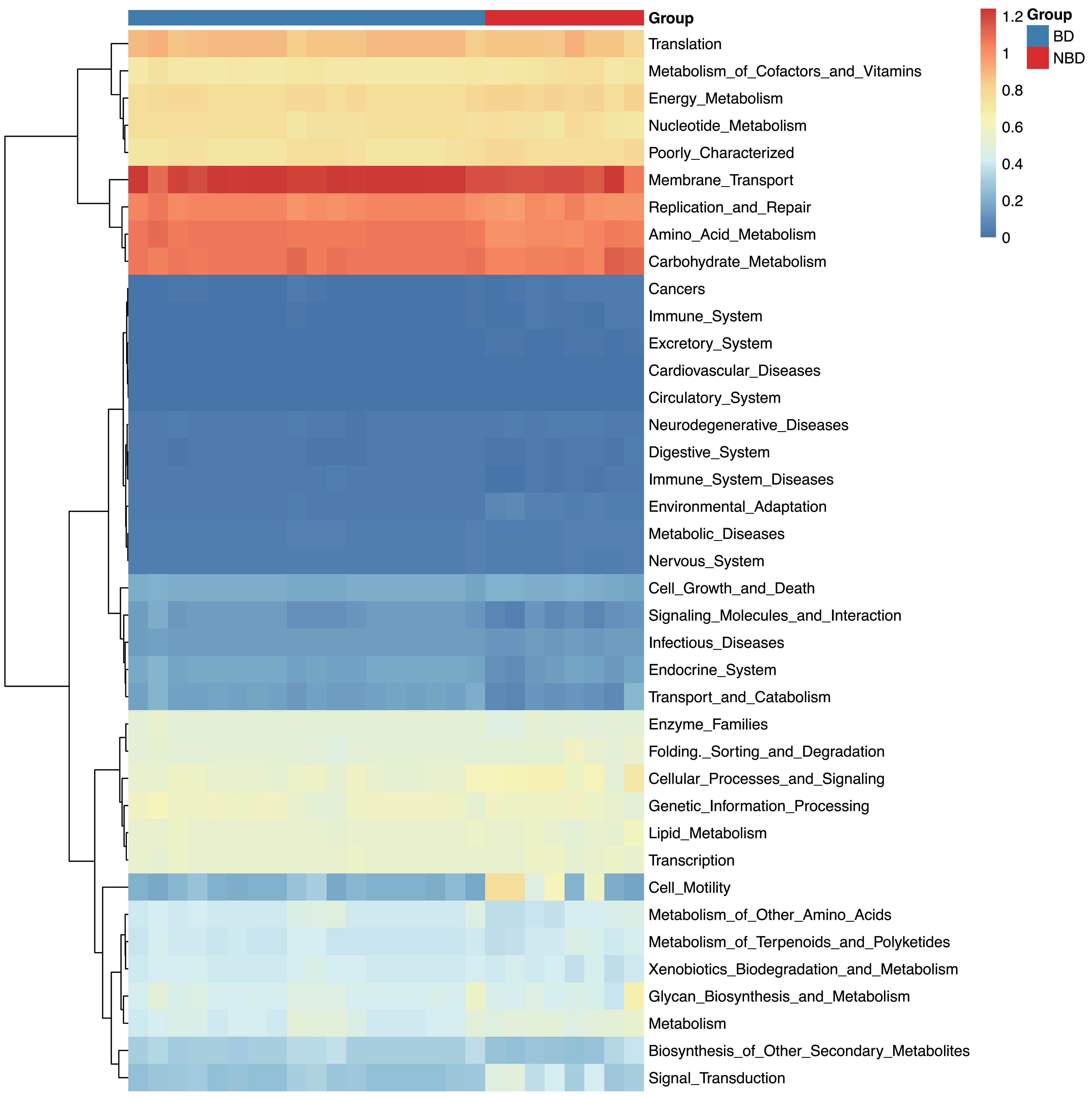

Group
Translation
Metabolism_of_Cofactors_and_Vitamins
Energy_Metabolism
Nucleotide_Metabolism
Poorly_Characterized
Membrane_Transport
Replication_and_Repair
Amino_Acid_Metabolism
Carbohydrate_Metabolism
Cancers
Immune_System
Excretory_System
Cardiovascular_Diseases
Circulatory_System
Neurodegenerative_Diseases
Digestive_System
Immune_System_Diseases
Environmental_Adaptation
Metabolic_Diseases
Nervous_System
Cell_Growth_and_Death
Signaling_Molecules_and_Interaction
Infectious_Diseases
Endocrine_System
Transport_and_Catabolism
Enzyme_Families
Folding._Sorting_and_Degradation
Cellular_Processes_and_Signaling
Genetic_Information_Processing
Lipid_Metabolism
Transcription
Cell_Motility
Metabolism_of_Other_Amino_Acids
Metabolism_of_Terpenoids_and_Polyketides
Xenobiotics_Biodegradation_and_Metabolism
Glycan_Biosynthesis_and_Metabolism
Metabolism
Biosynthesis_of_Other_Secondary_Metabolites
Signal_Transduction
1.2
1
0.8
0.6
0.4
0.2
0
Group
BD
NBD

C

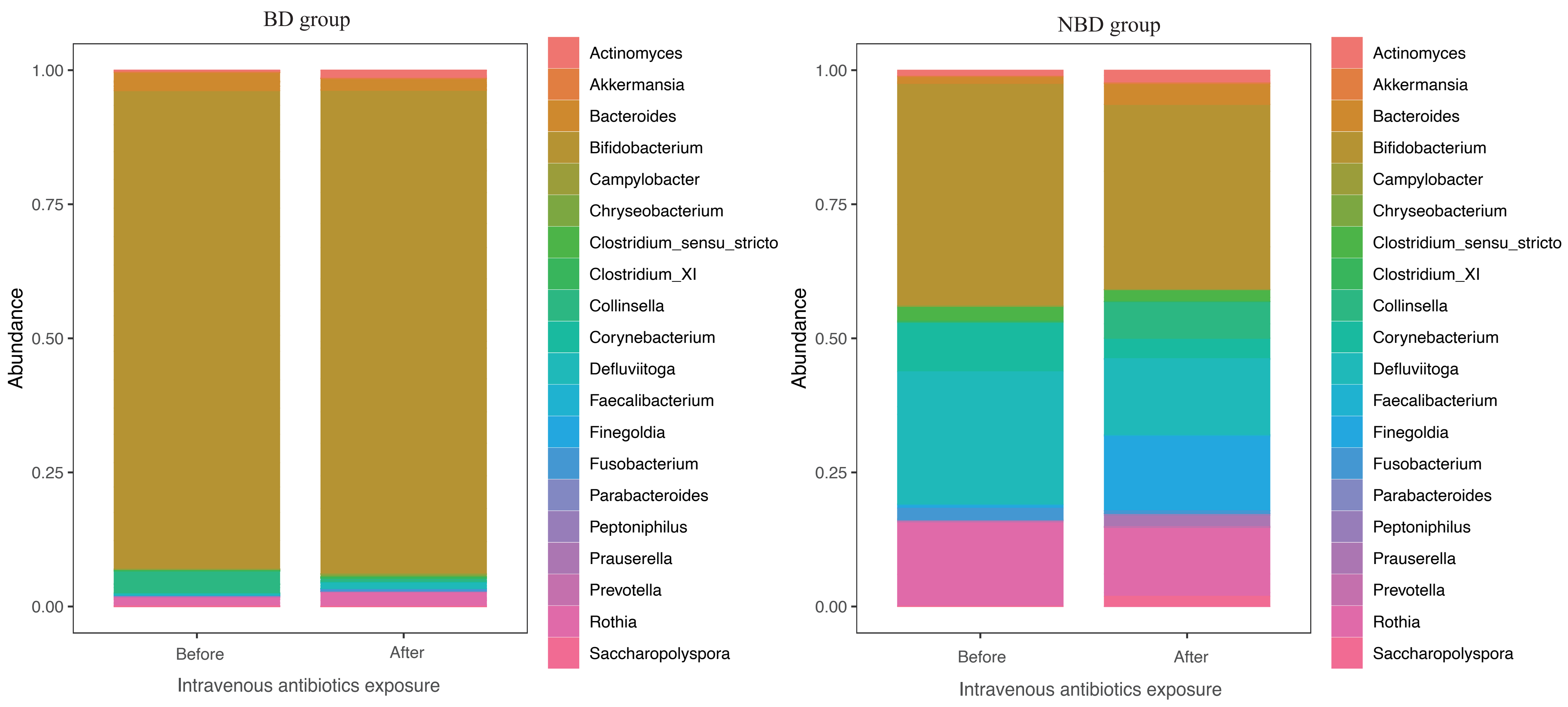

BD group
NBD group
1.00
0.75
0.50
0.25
0.00
Abundance
Before
After
Intravenous antibiotics exposure
Actinomyces
Akkermansia
Bacteroides
Bifidobacterium
Campylobacter
Chryseobacterium
Clostridium_sensu_stricto
Clostridium_XI
Collinsella
Corynebacterium
Defluviitoga
Faecalibacterium
Finegoldia
Fusobacterium
Parabacteroides
Peptoniphilus
Prauserella
Prevotella
Rothia
Saccharopolyspora

D

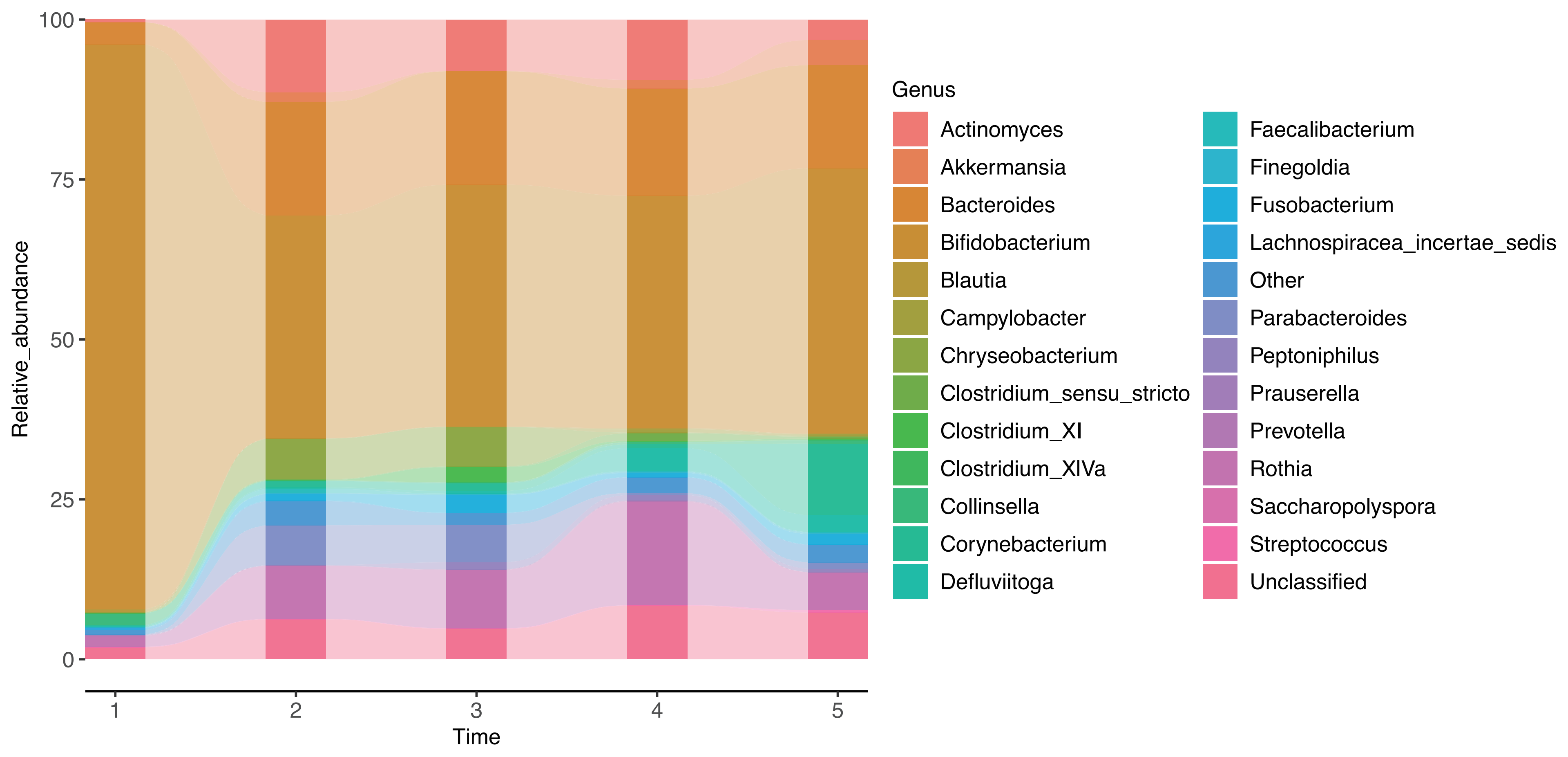

100
75
50
25
0
Relative_abundance
1
2
3
4
5
Time
Genus
Actinomyces
Akkermansia
Bacteroides
Bifidobacterium
Blautia
Campylobacter
Chryseobacterium
Clostridium_sensu_stricto
Clostridium_XI
Clostridium_XIVa
Collinsella
Corynebacterium
Defluviitoga
Faecalibacterium
Finegoldia
Fusobacterium
Lachnospiracea_incertae_sedis
Other
Parabacteroides
Peptoniphilus
Prauserella
Prevotella
Rothia
Saccharopolyspora
Streptococcus
Unclassified

E

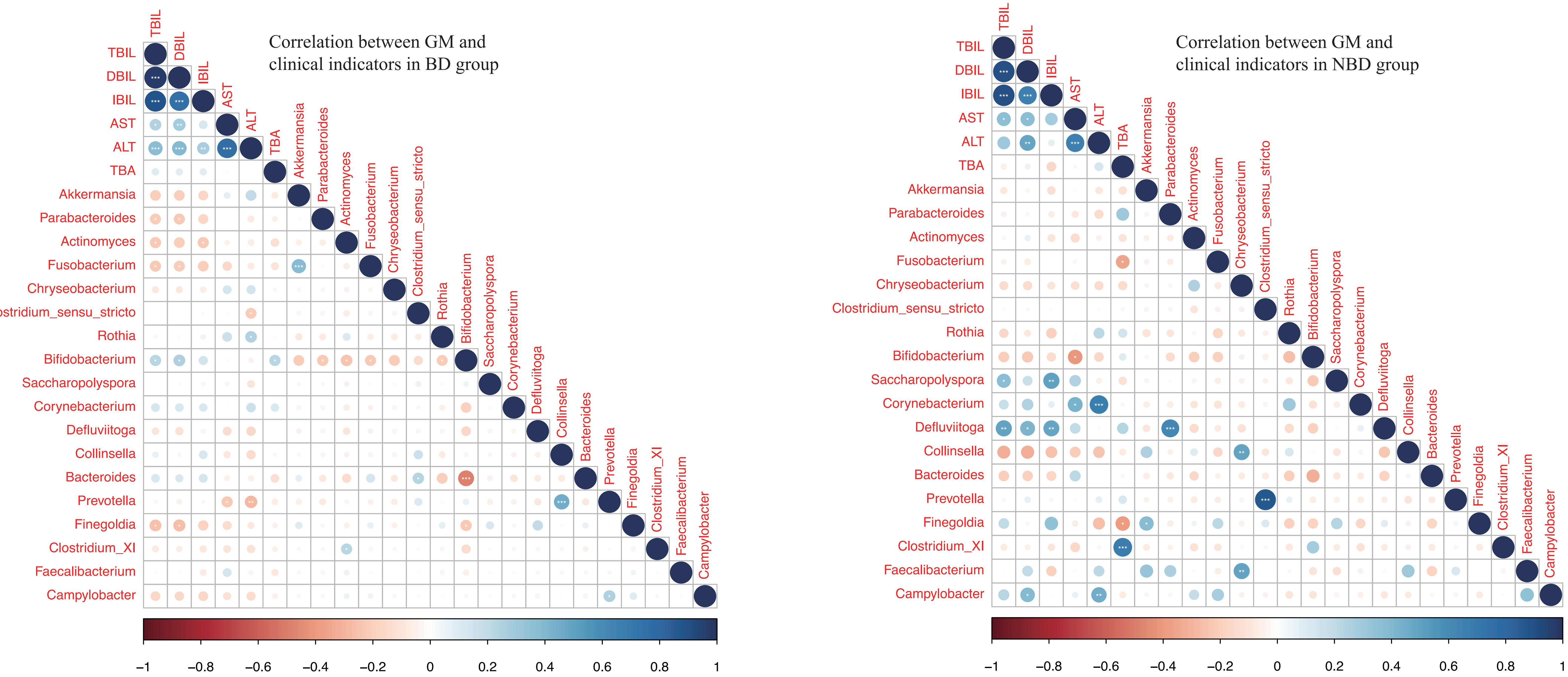
Correlation between GM and clinical indicators in BD group
Correlation between GM and clinical indicators in NBD group
TBIL
DBIL
IBIL
AST
ALT
TBA
Akkermansia
Parabacteroides
Actinomyces
Fusobacterium
Chryseobacterium
Clostridium_sensu_stricto
Rothia
Bifidobacterium
Saccharopolyspora
Corynebacterium
Defluviitoga
Collinsella
Bacteroides
Prevotella
Finegoldia
Clostridium_XI
Faecalibacterium
Campylobacter
−1
−0.8
−0.6
−0.4
−0.2
0
0.2
0.4
0.6
0.8
1

F

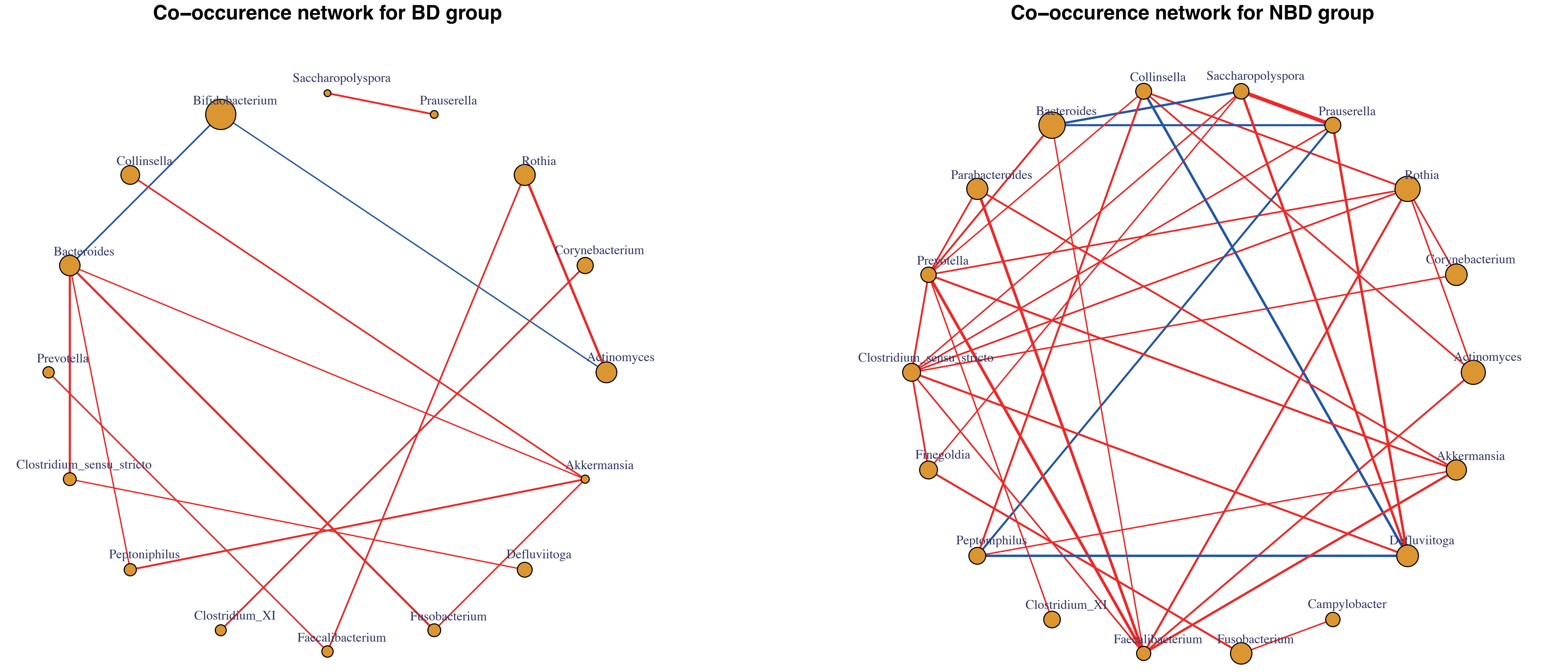
Co−occurence network for BD group
Co−occurence network for NBD group
Saccharopolyspora
Prauserella
Bifidobacterium
Collinsella
Rothia
Bacteroides
Corynebacterium
Prevotella
Actinomyces
Clostridium_sensu_stricto
Akkermansia
Peptoniphilus
Defluviitoga
Clostridium_XI
Fusobacterium
Faecalibacterium
Parabacteroides
Finegoldia
Campylobacter
